# Facial cutaneous Rosai-Dorfman disease: A case report and literature review

**DOI:** 10.3892/etm.2015.2260

**Published:** 2015-02-05

**Authors:** SHENG FANG, AI-JUN CHEN

**Affiliations:** Department of Dermatology, The First Affiliated Hospital of Chongqing Medical University, Chongqing 400016, P.R. China

**Keywords:** histiocytosis, lymphadenopathy, Rosai-Dorfman, cutaneous

## Abstract

Rosai-Dorfman disease (RDD), otherwise known as sinus histiocytosis with massive lymphadenopathy, is a rare disease. Cutaneous RDD (CRDD) is an extremely rare form of RDD, which is limited to the skin. The present study examined a case of purely CRDD in a 25-year-old female patient who presented with a two-month history of red plaques on her face. In addition, a review of the literature was conducted, where the etiology, pathology, clinical characteristics and treatment of the disease were discussed. From a dermatological perspective, the current study aimed to emphasize the histological features and clinical morphology of cutaneous RDD. Clinicians should have sufficient knowledge to be able to recognize and manage this rare condition. The present study found that the presence of reddish-yellow nodules on the face without any particular sensitivity may be useful in the diagnosis of CRDD. Treatment with topical steroids was found to be beneficial in alleviating CRDD.

## Introduction

Rosai-Dorfman disease (RDD), originally known as sinus histiocytosis with massive lymphadenopathy, is a non-Langerhans cell histiocytosis that was first described in 1965 by Destombes (1) and subsequently recognized as a distinct entity by Rosai and Dorfman in 1969 (2). There are two main forms of RDD: One form that affects the lymph nodes and in certain cases the extranodal organs (3), and the other form is purely cutaneous RDD (CRDD) (4). CRDD is extremely rare, with only a few reported cases. The etiology of CRDD remains unknown with a number of viral and immune causes hypothesized. CRDD presents as solitary or numerous papules, nodules, plaques or as a combination of these. Based on previous case reports, certain treatment options, including dapsone, thalidomide and isotretinoin, have been suggested for patients with varying efficacy (5–7). The present study reports a case of purely CRDD and discusses the clinical and histological features, etiology and course in conjunction with the literature review.

## Case report

**Figure 1:**
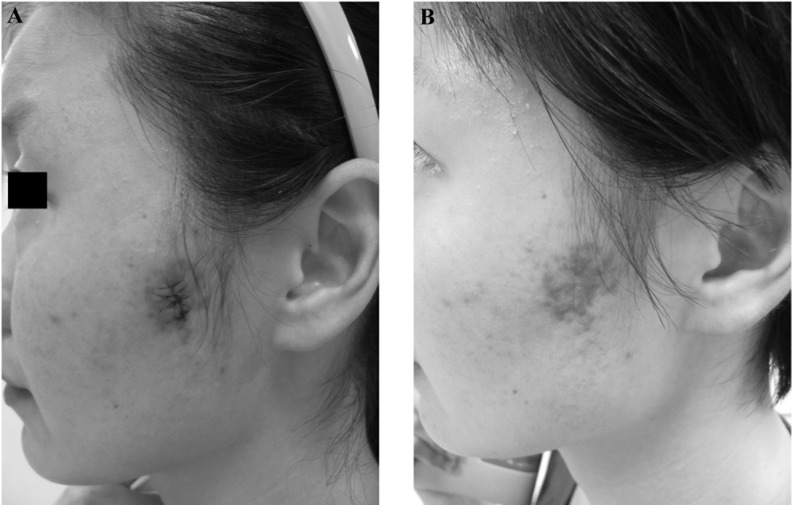
Patient with erythematous plaques and facial reddish-yellow nodules (A) prior to and (B) two months following treatment.

A 25-year-old Chinese female patient presented with a two-month history of red plaques on her face. The lesion grew progressively in size, with occasional pain and pruritus. The patient had no particular previous medical history, and was otherwise healthy, with no history of fever, malaise or weight loss. There was no mucosal involvement. The results of a general physical examination were normal, with no signs of lymph node enlargement. However, a dermatological examination showed indurated erythematous plaques with a number of reddish-yellow nodules on the left side of the patient’s face (Fig. 1).

**Figure 2:**
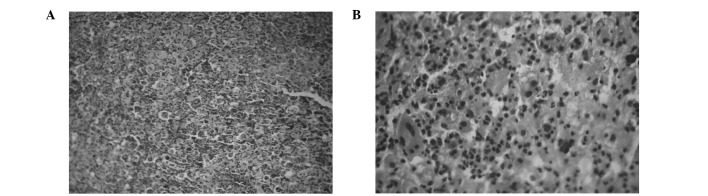
(A) Sheets of inflammatory infiltrates composed of neutrophils, plasma cells, lymphocytes and histiocytes in the dermis (hematoxylin and eosin; magnification, ×40). (B) Rosai-Dorfman cells showing cytophagocytosis of the lymphocytes and plasma cells (hematoxylin and eosin; magnification, ×400).

**Figure 3:**
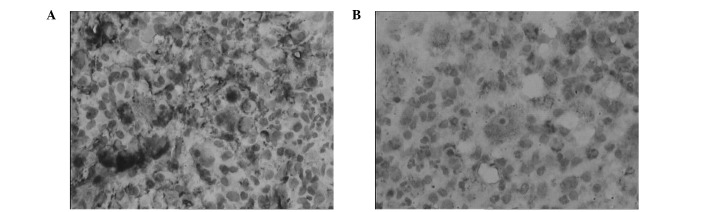
Immunohistochemical staining revealed the histiocytes were (A) strongly positive for S100 protein and (B) weakly positive for CD68 protein (SP staining; magnification, ×400).

The biopsy specimen obtained from the afflicted area showed that the epidermis was normal, but revealed dense inflammatory infiltrates, composed of neutrophils, plasma cells, lymphocytes and histiocytes, in the dermis. No significant histiocytic atypia was identified. The histiocytes exhibited abundant, focally foamy cytoplasm and large vesicular nuclei; lymphocytes and plasma cells were observed within the cytoplasm of these histiocytes (emperipolesis; Fig. 2). Immunohistochemical staining revealed that the histiocytes were strongly positive for S100 protein, weakly positive for CD68 and negative for CD1a (Fig. 3). Thus, the diagnosis of CRDD was confirmed. Laboratory examination revealed no abnormality in routine tests, including the blood count, urinalysis, erythrocyte sedimentation rate, C-reactive protein levels, liver and kidney function tests and muscle enzymes. In addition, the serum protein level and collagen vascular screening tests (antinuclear antibodies, C3, C4) were normal.

The patient was treated with a twice-daily topical application of mometasone furoate 0.1% cream for one month. In addition, local injections of 1 mg compound betamethasone were administered once a month for two months. The plaque treated with local injections of the steroid showed improvement. However, the patient declined to continue with the injection therapy due to side-effects, including increased blood pressure and weight gain, from the systematic use of corticosteroids. The patient showed slight improvement in the three months of outpatient follow-up which involved a telephone call every month. Written informed consent was obtained from the patient prior to participation in the present study.

## Discussion

The term CRDD is used to describe the forms of RDD that only involve the skin. CRDD differs from RDD in that RDD exhibits systemic involvement (4,8). Extranodal forms occur in 43% of RDD cases, with the skin being the most common site. Approximately 10% of RDD patients exhibit skin lesions, and in 3% of cases, the disease presents exclusively in the skin (5). Purely CRDD was reported for the first time by Thawerani *et al* (4) in 1978 in a 48-year-old male patient who presented with a solitary nodule on the shoulder. Since then, a number of cases of CRDD have been reported (8,9). It has been suggested that the CRDD and RDD variants of the disease are distinct clinical entities (10). The purely cutaneous form of RDD, as observed in the patient of the present study, is very rare. CRDD has a later age of onset (median age, 43.5 years) compared with RDD. In addition, CRDD shows a marked female predominance (2:1) and most commonly affects Asian and Caucasian individuals. By contrast, RDD has a median onset age of 20.6 years and is slightly more common in males (1.4:1). The majority of RDD patients are of African descent and the disease is rarely reported in Asian patients (10).

The etiology of CRDD remains unknown with viral and immune causes hypothesized. The polyclonal nature of the cell infiltrate and the clinical progression of RDD suggest a reactive process rather than a neoplastic disorder (11). The cell of origin in RDD is uncertain; however, Middel *et al* found that stimulation of monocytes and macrophages via macrophage colony-stimulating factor generated immunosuppressive macrophages, which may represent the primary mechanism for the pathogenesis of RDD (12). An additional study by Mannan and Karak proposed a dendritic cell origin (13). In certain cases, human herpesvirus (HHV)-6 and Epstein-Barr virus infections were found to be associated with the pathogenesis of RDD (14). Luppi *et al* identified HHV-6 viral antigen expression in two cases of RDD (15). Furthermore, Levine *et al* used *in situ* hybridization to detect HHV-6 in the tissues of RDD patients (14). Parvovirus B19 has also been identified in four RDD patients in an immunohistochemical study (16).

Histological findings in CRDD are usually similar to those in RDD. Typically, the epidermis is normal. In the dermis, a diffuse infiltrate of histiocytes is accompanied by a background infiltrate of lymphocytes and plasma cells. Lymphoid follicles with reactive germinal centers may also be present. The phenomenon of emperipolesis, which represents the presence of intact lymphocytes within histiocytes, is common in CRDD. Less often, the cytoplasm may contain plasma cells, neutrophils and red blood cells. Mitoses and nuclear atypia are rare, while necrosis is absent. CRDD histiocytes stain positively for S100 protein and CD68, but negatively for CD1a, which can be used to confirm the diagnosis of CRDD. Electronic microscopy of CRDD tissue reveals no signs of Birbeck granules, which eliminates the possibility of Langerhans cell histiocytosis (17).

Despite the distinctive histological features, the clinical diagnosis of CRDD is hard to confirm, as the clinical presentation is variable in the absence of lymphadenopathy. The lesions in CRDD, which may be solitary or numerous, usually present as papules, nodules, plaques or as a combination of these (8,9). In certain cases, the lesions may present as pustules, acneiform lesions and lesions mimicking vasculitis and panniculitis, or even a breast mass (18). The patient in the present study exhibited a facial profusion of indurated erythematous plaques with a number of reddish-yellow nodules. The most common site of lesions in CRDD is the face, followed by the back, chest, thigh, flank and shoulder. The prognosis in CRDD is reasonably good; however, the condition may be associated with the involvement of other disorders, including bilateral uveitis, antinuclear antibody positive lupus erythematosus, rheumatoid arthritis, hypothyroidism, lymphoma and HIV infection (19,20).

The treatment of CRDD is challenging. Numerous treatment methods have been attempted; however, an ideal option has not been identified in all cases and the response is often poor. As RDD is characterized as a benign and self-limiting disease, it has been suggested that less aggressive therapeutic approaches should be used if possible. Surgical excision of the lesions has been helpful in certain cases. Cryotherapy and local radiation have also been found to improve the condition. In addition, dapsone and thalidomide have been effective in cases refractory to other treatments (5,6). Mixed results have been observed with isotretinoin and imatinib (7,21); a number of patients improved, while the condition in other patients remained poor. Utikal *et al* described a patient with complete remission of CRDD after receiving imatinib therapy (21); however, a different study reported a patient with CRDD who was completely resistant to this treatment (22). The patient in the present study was treated with topical steroids and showed improvement. However, the patient decided to discontinue treatment due to side-effects.

In conclusion, CRDD is an unusual clinical entity comprising a wide-spectrum of lesions, which vary clinically and histologically. The clinical morphology is variable; however, often histological features may be characterized to aid the confirmation of a diagnosis. Generally, CRDD follows a benign clinical course, with a possibility of spontaneous remission. However, further molecular and genetic studies are required to explain the predominant involvement of skin and the higher incidence of the disease in Asian patients.
